# Transcriptomic Analyses Reveal Gene Expression Profiles and Networks in Nasopharyngeal Carcinoma

**DOI:** 10.1155/2021/8890176

**Published:** 2021-01-25

**Authors:** Yaqi Zhou, Weiqiang Yang, Xueshuang Mei, Hongyi Hu

**Affiliations:** ^1^Peking University Shenzhen Hospital, Shenzhen Peking University-The Hong Kong University of Science and Technology Medical Center, Shenzhen, Guangdong Province, China; ^2^Department of Otorhinolaryngology, Peking University Shenzhen Hospital, Shenzhen, Guangdong Province, China

## Abstract

**Background:**

Nasopharyngeal carcinoma (NPC) is a rare but highly aggressive tumor that is predominantly encountered in Southeast Asia and China in particular. Aside from radiotherapy, no effective therapy that specifically treats NPC is available, including targeted drugs. Finding more sensitive biomarkers is important for new drug discovery and for evaluating patient prognosis.

**Methods:**

mRNA expression datasets from the Gene Expression Omnibus database (GSE53819, GSE64634, and GSE40290) were selected. After all samples in each dataset were subjected to quality control using principal component analyses, the qualified samples were used for additional analyses. The genes that were significantly expressed in each dataset were intersected to identify the most significant of these. Gene functional enrichment analyses were performed on these genes, using Gene Ontology (GO) and Kyoto Encyclopedia of Genes and Genomes analyses. The protein–protein interaction network of selected genes was analyzed using the Search Tool for the Retrieval of Interacting Genes database. Significantly, differentially expressed genes were further verified with two RNA-seq datasets (GSE68799 and GSE12452), as well as in clinical samples.

**Results:**

In all, 34 **(**8 upregulated genes and 26 downregulated) genes were identified as significantly differentially expressed. The immune response and the regulation of cell proliferation were the most enriched biological GO terms. Using reverse transcription quantitative real-time PCR (RT-qPCR), the genes MMP1, AQP9, and TNFAIP6 were detected to be upregulated, and FAM3D, CR2, and LTF were downregulated in NPC tissue samples.

**Conclusion:**

This study provides information on the genes that may be involved in the development of NPC and suggests possible druggable targets and biomarkers for diagnosing and evaluating the prognosis of NPC.

## 1. Introduction

Nasopharyngeal carcinoma (NPC), which originates in the nasopharynx epithelium, is often observed in the pharyngeal recess. NPC is an aggressive head and neck cancer that is highly prevalent in Southeast Asia, particularly in South China [1]. This distinct geographical distribution aligns with other features that distinguish it from other head and neck cancers [2]. Radiotherapy is the main method used to treat NPC, but recurrence after primary radiotherapy, with or without chemotherapy, and treatment failure is severe, at a rate of around 10–30% [3, 4]; this has prompted researchers to seek novel effective therapies, such as a targeted drug therapy that could be combined with radiotherapy.

Studying changes in the gene expression in NPC is an important way to identify biomarkers of early clinical diagnosis, improve evaluation of prognosis, and most importantly, target molecules that can effectively target NPC. High-throughput sequencing allows for the identification of profiles of gene sets that are changed in cancerous tissues relative to normal controls. A combination of multiple microarray studies and RNA-seq studies could help us rule out bias from a single sequencing run and together can provide us with more solid and reliable results to ground further investigation.

Previous studies have reported single transcriptome analyses of NPC or reanalyses of a few integrated datasets [5–7]. In our study, we examined changes in the mRNA expression. To investigate mRNA expression profiles in NPC, we searched the Gene Expression Omnibus (GEO) database [8], obtained all the files relevant to NPC, and reviewed each by case numbers and methods. We excluded records for which the original annotation files are not available. Using strict quality control enabled by principal component analysis (PCA), the three RNA microarray datasets GSE64634, GSE53819, and GSE40290 were used in our primary analyses as discovery sets, including 42 NPC tumor tissues and 14 normal tissue controls in total. We conducted downstream analyses, including functional enrichment and protein–protein interaction analyses, of 34 significantly differentially expressed genes (DEGs). Then, the two datasets GSE68799 and GSE12452 were selected as verification sets to validate these DEGs. Clinical samples were also taken for verification.

## 2. Materials and Methods

### 2.1. Microarray Studies, Data Sets, and Sample Characteristics from the GEO Database

The GEO database was used to search all publicly available data about NPC. Each study in the database was reviewed for whether it met the following criteria: data are from the mRNA expression sequencing or microarray analyses of NPC; datasets are available for download and reanalysis, and data include an NPC sample and a control sample, as well as detailed information on the technique and platform used for each. Following these criteria, six datasets were included in our primary study.

### 2.2. Differential Expression Analyses and Data Visualization

Next, differential analyses were performed to compare tumor tissues to normal tissues using GEO2R for each revised microarray analysis and the DESeq2 package for the RNA-seq data in the *R* computing environment. Gene lists were filtered by ∣log_2_FC | >2 and *p* < 0.05 and then were intersected together. Volcano plots were used to visualize the DEGs.

### 2.3. Gene Ontology and KEGG Pathway Analyses, Functional Enrichment Analyses, and Protein–Protein Interaction

Gene ontology (GO) and KEGG pathway enrichment analyses were performed using the Database for Annotation, Visualization, and Integrated Discovery (DAVID, version 6.7). Protein–protein interaction analyses were performed using the database of the Search Tool for the Retrieval of Interacting Genes (STRING), version 11.0 [9], with a medium confidence ≥ 0.4. Genes were clustered with k-means clustering.

### 2.4. Clinical Sample Collection and Total RNA Preparation

This study was approved by the Research Ethics Committee of Peking University Shenzhen Hospital, and written informed consent was obtained from all patients. Three NPC tissues and three normal nasopharynx tissues were obtained from each patient who had undergone a nasopharynx biopsy from December 2017 to December 2019 at the Peking University Shenzhen Hospital, China. The tissue samples were stored at -80°C in a freezer until total RNA was extracted using a total RNA extraction kit (BioTeke, China, code no: RP1201).

### 2.5. RT-qPCR Analyses

RT-qPCR was conducted as previously described [10], and 1 *μ*g total RNA was reverse transcribed in a 20 *μ*L reaction system using StarScript II First-strand cDNA Synthesis Mix with gDNA Eraser (GenStar, China, code no. A224-10) following the manufacturer's instructions. The reaction products were diluted in 80 *μ*L distilled water. The real-time PCR reaction was performed with 1 *μ*L diluted reverse transcription product, 5 *μ*L TB Green™ Premix Ex Taq™ II (Takara Bio Inc., code no, RR820A), and 0.4 *μ*L forward and 0.4 *μ*L reverse primers (0.4 *μ*M). The reaction was performed in a LightCycler 96 Sequence Detection System (Roche, Basel, Switzerland) for 40 cycles (95°C for 5 s, 55°C for 30 s, and 72°C for 30 s) after an initial 30 s denaturation at 95°C. GAPDH was used as an internal control. The RNA levels of the tumor samples and control samples were calculated using the 2^–*Δ*Ct^ method. All primers of the hub genes and GAPDH were synthesized by Sangon Biotech (Shanghai, China), and the sequences are listed in Table 1.

### 2.6. Statistical Analyses

The statistical analyses were performed using Prism GraphPad 8.4.0 software (San Diego, CA). In the RT-qPCR analyses, the results from the NPC samples and control samples were compared using Student's *t*-test, with a significance threshold of *p* < 0.05.

## 3. Results

### 3.1. Strategy of Transcriptome Biostatistical Analyses in NPC

**Figure 1:**
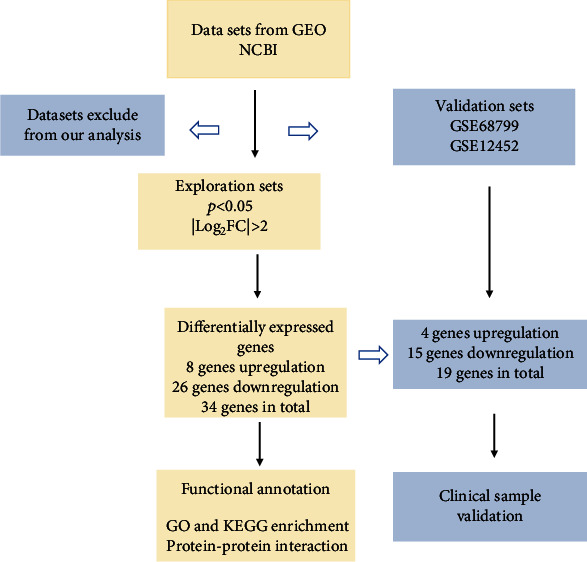
Scheme of the bioinformatic analyses in NPC datasets from the GEO database.

To select all available NPC transcriptome data, the GEO database was used. After reviewing each possible dataset, we selected six from which the primary data could be obtained and analyzed. PCAs were first used to check the quality of each set. After all unqualified files were excluded, gene expression analyses were performed separately in each dataset. A differentiated hub gene list was created from the intersection of three datasets, and gene functional annotations and assessment of protein–protein interactions were performed. In addition, we intersected all the genes separately with two RNA-seq datasets and selected a few for validation in clinical NPC samples. The complete scheme of this study is given in Figure 1.

### 3.2. PCAs to Verify the Group Independence of each Dataset

**Figure 2:**
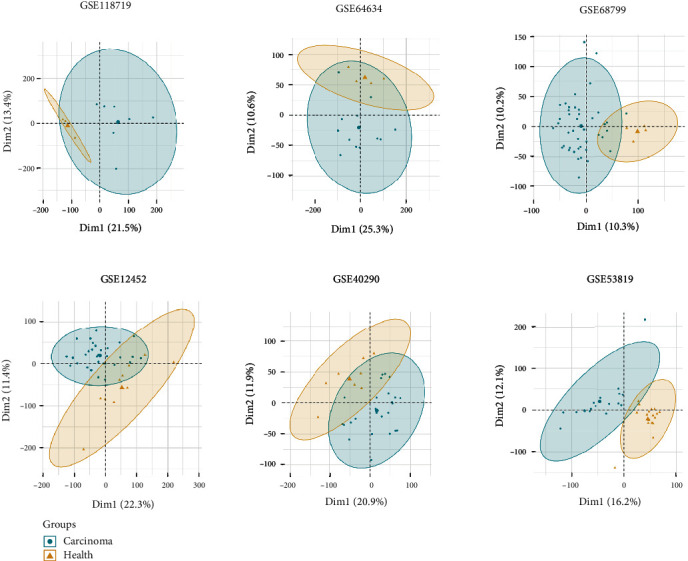
Principle component analyses of all datasets selected from the GEO database. Two-dimensional plots of normal and tumor groups with the top two principal components (Dim1 and Dim2). The horizontal and vertical axes represent the distribution of each sample within Dim1 and Dim2, respectively.

To analyze the differences between the control and NPC group, we performed PCAs of the dataset from the GEO database, which demonstrated the independence of each group. The results, presented in Table 2, include NPC samples and control samples for all selected datasets, including GSE118719, GSE68799, GSE53819, GSE12452, GSE40290, and GSE64634. GSE118719 showed a crossover between NPC and control groups, and it has fewer samples than any other dataset, so we excluded it from our analyses (Figure 2).

**Figure 3:**
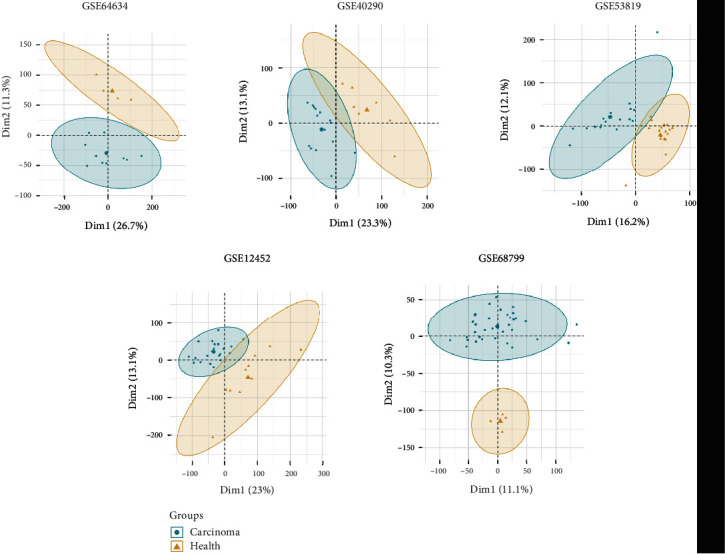
Principle component analyses of revised datasets from which the unqualified files were removed. Two-dimensional plots of normal and tumor groups with the top two principal components (Dim1 and Dim2). The horizontal and vertical axes represent the distribution of each sample within Dim1 and Dim2, respectively.

For the remaining datasets, tumor samples that were close to the control samples were excluded for the next step (X740T, X707T, X756T, X701T, X723T, X728T, X737T, GSM312923, GSM312907, GSM312935, GSM312926, GSM312936, GSM312911, GSM312913, GSM312915, GSM312917, GSM312930, GSM1575903, GSM1575905, GSM990733, GSM990753, GSM990752, GSM990736, GSM990744, GSM990735, GSM990734, GSM990741, GSM990745, GSM990747, and GSM990757) (Figure 3).

### 3.3. Identification of Significantly Differentially Expressed Genes Related to NPC

**Figure 4:**
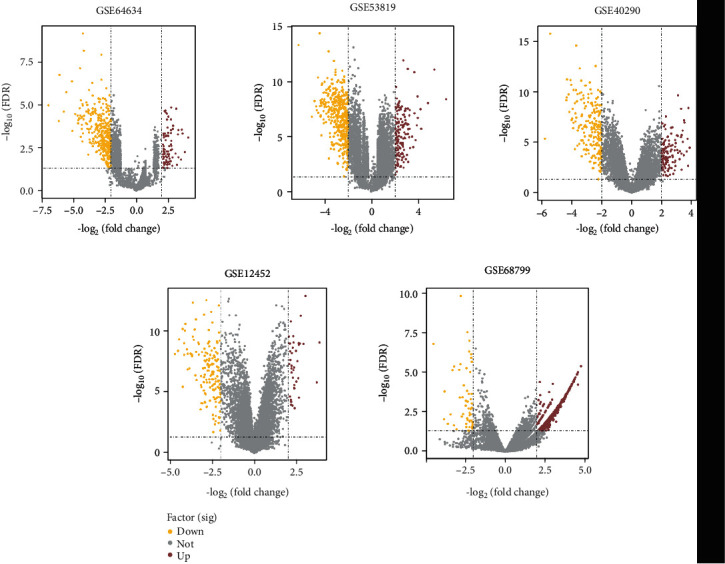
Differentially expressed genes in all five datasets from the GEO database. Volcano plots show the number of differentially expressed genes identified from each of the five GEO datasets. Yellow dot, downregulated genes; Brown dot, upregulated genes.

**Figure 5:**
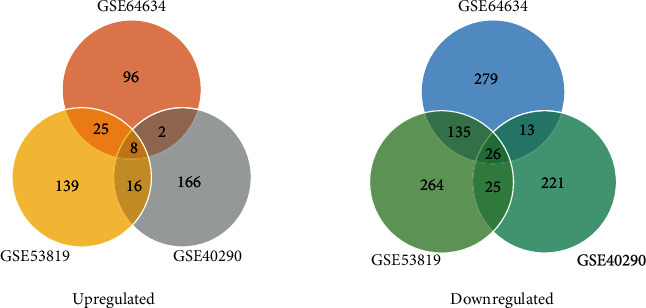
Differentially expressed genes in three datasets from the GEO database. Upregulated and downregulated genes in each dataset were intersected separately, and 8 upregulated genes and 26 downregulated genes were identified.

For each dataset, genes with *p* < 0.05 and ∣log2fold change | >2 were identified as significantly changed genes. A volcano plot was used to exhibit the DEGs in each dataset (Figure 4). In all, three RNA microarray datasets (GSE53819, GSE64634, and GSE40290) were used as an exploratory set to filter out the DEGs related to NPC. In our primary analyses, 42 NPC tumor tissues and 14 normal controls were used. The datasets were analyzed by intersection, and 8 upregulated and 26 downregulated genes were identified as significantly differentially expressed in NPC tissues relative to normal controls (Figure 5).

### 3.4. GO Functional Annotation and KEGG Pathway Analyses of all Selected Genes

**Figure 6:**
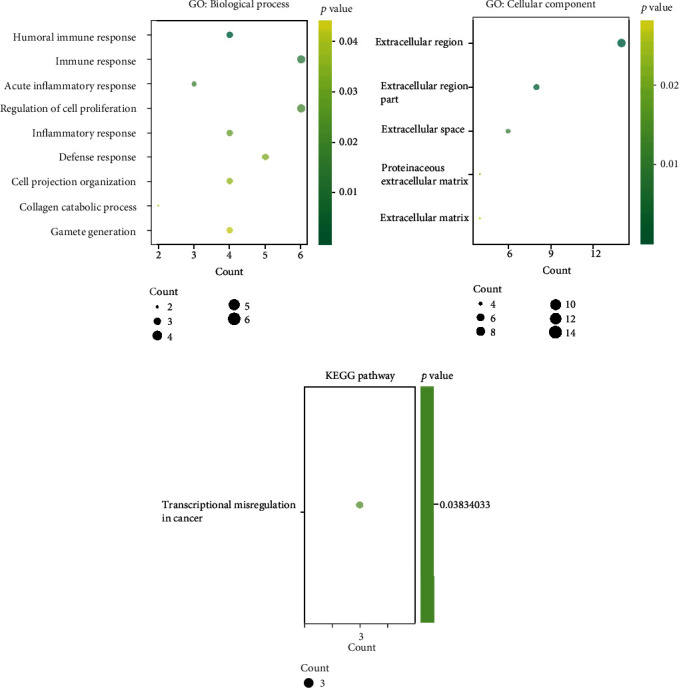
GO annotations and KEGG pathway functional enrichment analyses of all differentially expressed genes in our study. The bubble plots show the GO and KEGG pathway enrichment data, and the *X*-axis shows the gene numbers, and the color indicates the *p* value.

GO enrichment analyses were performed on the 34 significantly DEGs. The most enriched GO terms included the humoral immune response, regulation of cell proliferation, and others (Figure 6). KEGG pathway analyses showed one enrichment pathway, namely, transcriptional misregulation in cancer, which included the three genes HOXA10, MMP3, and PROM1.

### 3.5. Protein–Protein Interaction Network of All Selected Genes

**Figure 7:**
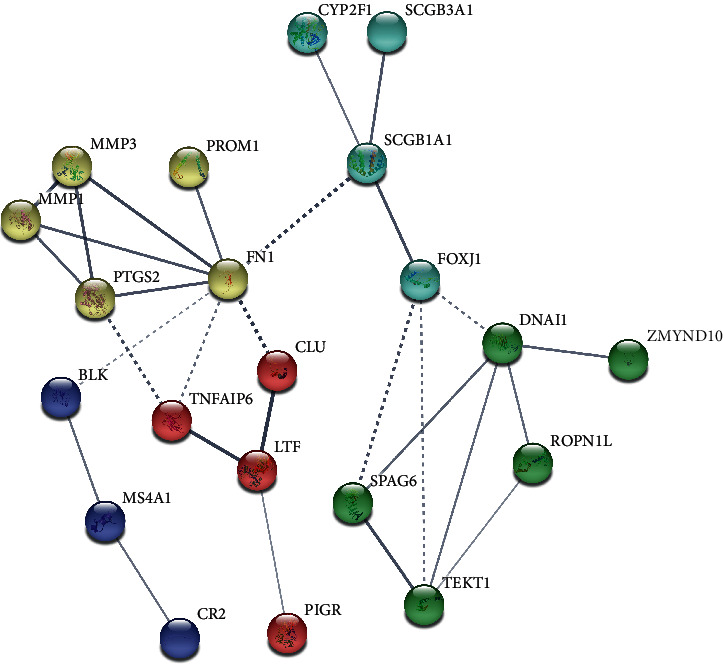
The protein–protein interaction network indicates the main clusters of differentiated proteins. The thickness of the line between two nodes indicates the strength of the supporting data. Different colors indicate different groups according to Gene Ontology functional clustering.

To explore the protein–protein interactions among all the genes selected from the database, the STRING platform was used. The resulting network is shown in Figure 7, where all proteins are grouped into five clusters by k-means clustering, which indicates the possible protein interaction network that could be involved in the development of NPC.

### 3.6. Verification of DEGs by Validation Sets

Then, 34 genes were further analyzed in two datasets acquired from the GEO database. These datasets both contain RNA-seq data from the Illumina HiSeq 2000 platform. After the intersection, two upregulated genes and one downregulated gene were identified in GSE68799, and four upregulated and 15 downregulated genes were found in GSE12452 (Table 3).

### 3.7. The Expression Level of Select Genes in Clinical NPC Samples

**Figure 8:**
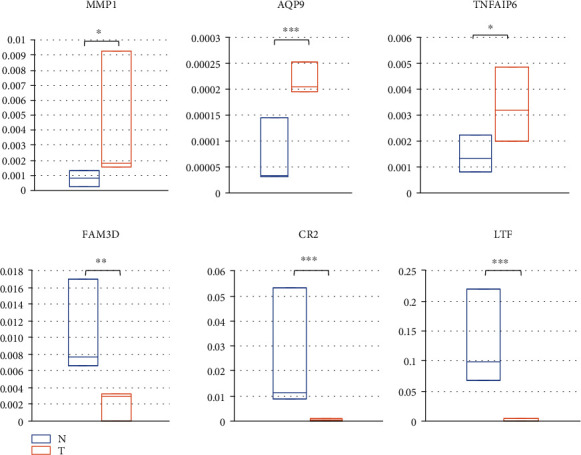
The mRNA expression level for six hub genes in NPC tissues relative to the normal nasopharynx tissue. Quantitative RT-PCR was used in this analysis. A *t*-test was performed to determine the differences between the groups. N: normal control; T: NPC tumor tissues; ^∗^*p* < 0.05, ^∗∗^*p* < 0.01, ^∗∗∗^*p* < 0.001.

Six DEGs were selected for our further analyses in patient samples (including three NPC tissue samples and three normal control nasopharynx tissues). MMP1 and LTF were selected as known up- and downregulated genes in NPC. As shown in Figure 8, MMP1, AQP9, and TNFAIP6 were overexpressed in tumors compared to controls, and FAM3D, CR2, and LTF were downregulated in NPC.

## 4. Discussion

NPC is a severe cancer found particularly in South China. Its sensitivity to radiotherapy is a hopeful sign for NPC patients, but its high recurrence rate requires more effective methods of targeting for NPC treatment. In our study, we gathered all available RNA-seq and RNA microarray data to select possible biomarkers for NPC, and we also obtained information to study the etiology of NPC and its possible molecular signal transduction pathway during tumorigenesis in greater detail.

PCAs were first performed to control data quality, as they provide a method of simplifying complexity in high-dimensional data and emphasizing variation [11]. After PCAs, we excluded the unqualified samples and analyzed the remaining samples in each dataset separately, considering that the integration of different microarray platforms and analytical conditions could affect the results. Through our analyses, we obtained 34 significantly DEGs, including 8 upregulated and 26 downregulated ones. A few upregulated genes were previously reported as oncogenes and play important roles in the proliferation, progression, or metastasis of different cancers. Some downregulated genes act as tumor suppressors in many cancers.

PTGS2 (COX-2) encodes cyclooxygenase 2, an important enzyme for prostaglandin biosynthesis during inflammation or wound healing. Cox-2 is overexpressed in many cancers, including breast cancer, colorectal cancer, and NPC [12–14]. It is also a biomarker for poor NPC prognosis [15].

Matrix metalloproteinase 1 and 3 (MMP1 and MMP3), which are in the matrix metalloproteinase family, are also overexpressed in many cancers. MMP-1 is overexpressed in breast cancer, colon cancer, and NPC [16, 17]. Its function in NPC has been well studied. The overexpression of MMP1 has been detected in NPC tissues and NPC cell lines, and the high MMP-1 expression significantly suppresses the sensibility of 5-FU chemotherapy in NPC [18]. The overexpression of MMP-3 is associated with metastasis in ductal breast cancer patients [19]. However, the function of MMP-3 in NPC has not yet been studied.

HOXA10 is another oncogene that is overexpressed in acute myeloid leukemia, NPC, and many other cancers [20, 21]. The high expression of HOXA10 promotes proliferation in bladder cancer, and it is also related to the low survival rate of bladder cancer patients [22]. The overexpression of HOXA10 induces proliferation and invasion in NPC cells [20].

Fibronectin 1 (FN1) belongs to the glycoprotein family. It is upregulated in many cancers, including NPC. The overexpression of FN1 in NPC cell lines promotes proliferation, migration, and invasion of NPC cells. It suppresses apoptosis in NPC cells by upregulating BCL-2 [23].

The remaining upregulated genes are also involved in tumor development, but their function in NPC remains unclear. TNFAIP6, also called TSG-6, belongs to the tumor necrosis factor alpha-induced protein family, and it may be related to cell migration. It is crucial for the maintenance of the stemness of murine mesenchymal stem cells [24, 25]. AQP9 is downregulated in hepatocellular carcinoma but is elevated in many other cancers, including clear cell renal cell carcinoma, which suggests that it plays a complex role in cancer development [26, 27]. A few bioinformatic studies have shown that COL8A1 is a hub gene associated with several cancers, but this requires further investigation.

Some genes that showed downregulation in our study might serve as tumor suppressors in NPC, and some of these have been studied in NPC. GTP-binding protein RAD (RRAD) has been reported to be a tumor suppressor in NPC, where the high methylation of the promoter region in RRAD causes its low level of expression and inactivates its function [28]. High methylation of the gene ZMYND10 has been reported in NPC and has been suggested to be a biomarker for NPC prognosis [29]. One study reported that serum levels of polymeric immunoglobulin receptor (PIGR) are downregulated in NPC patients, and low levels of the PIGR expression are strongly related to advanced clinical stages and to worse overall survival, making this useful as a prognosis biomarker. However, its expression level in the NPC tissue and function in the development of NPC remain unclear [30]. Lactotransferrin (LTF) has been reported to be downregulated in NPC tissues and acts as a tumor suppressor by repressing the AKT signaling pathway in NPC [31]. Some genes that we have identified here are also involved in the development of other cancers. Microseminoprotein beta (MSMB) has been reported to be downregulated in prostate cancer tissues and in patient serum [32]. The expression level of uroplakin-1b (UPK1B) is upregulated in bladder cancer, and it shows an oncogene function [33]. Forkhead box protein J1 (FOXJ1) is upregulated in bladder cancer and colorectal cancer [34, 35] but downregulated in ependymoma and choroid plexus tumors [36]. The downregulated genes that we have identified in NPC, such as MSMB, UPK1B, and FOXJ1, may be associated with the development or progression of NPC and may play different roles from their roles in other cancers.

We also validated six genes that were up- or downregulated in clinical NPC tissues using quantitative RT-PCR. MMP1 is a known upregulated gene that is associated with NPC. TNFAIP6 and AQP9 are newly identified genes that are overexpressed in NPC. Further studies are in the process in our group to identify how the overexpression of these two genes could regulate NPC development and progression. We validated only three of the downregulated genes. LTF was reported to be downregulated in NPC in a previous study [31], and FAM3D and CR2 are newly validated genes that may be involved in the development of NPC. Complement receptor type 2 (CR2) is an important receptor for primary infection of EBV in B cells and epithelial cells [37]; however, we found it to be downregulated in NPC, which suggests a different manner in which EBV can affect NPC. The CR2 promoter region also showed high DNA methylation rates [38], but its actual role in NPC remains unclear and requires deeper study. FAM3D belongs to the family with the sequence similarity 3 (FAM3) gene family, and it is overexpressed in colon cancer [39]. Further study is needed to investigate its function in NPC.

## 5. Conclusions

In summary, we identified 34 hub genes, including known and unknown genes associated with NPC. More functional studies must be performed to validate the hub genes identified from multiple transcriptome analyses. Our study provides information to continue to study the mechanism of development of NPC and more importantly to find biomarkers and druggable targets that will be useful for prognosis and new drug discovery.

## Figures and Tables

**Table 1:** Primer sequences used for quantitative real-time PCR.

Gene symbol	Primer sequence
GAPDH	F:5′-AACATCATCCCTGCCTCTACTGG-3′ R:5′-CCTCCGACGCCTGCTTCAC-3′
MMP1	F: 5′-ATGAAGCAGCCCAGATGTGGAG-3′ R: 5′-TGGTCCACATCTGCTCTTGGCA-3′
AQP9	F: 5′-CTGAACAGTGGCTGTGCCATGA-3′ R: 5′-CCACTACAGGAATCCACCAGAAG-3′
TNFAIP6	F: 5′-TCACCTACGCAGAAGCTAAGGC-3′ R: 5′-TCCAACTCTGCCCTTAGCCATC-3′
CR2	F: 5′-AGCCATCTGCACCAGTCTGTGA-3′ R: 5′-TCTTCTCCCACCAGCACATAGC-3′
FAM3D	F: 5′-CTACGACGATCCAGGGACCAAA-3′ R: 5′-CCTGAGGTCTTTGGCTCCTATG-3′
LTF	F: 5′-GGCTACTTCACTGCCATCCAGA-3′ R: 5′-ACTCCACTGGTTACACTTGCGC-3′

**Table 2:** Details of all datasets used and modified after selection from GEO database.

GSE	Publication	Upregulated genes	Downregulated genes	Platform	Sample size	Used as
GSE118719	*Journal of Experimental and Clinical Cancer Research*	4197	3226	Illumina HiSeq 4000	7 NPC 4 controls	Excluded

GSE53819	*Cell Cycle*	188	450	Affymetrix Human Genome U133 Plus 2.0 Array	18 NPC 18 controls	Analyzing set

GSE64634	*Oncotarget*	131	453	Affymetrix Human Genome U133 plus 2.0 Array	10 NPC 4 controls	Analyzing set

GSE40290		192	285	Capitalbio 22 K Human oligo array version 1.0	14 NK-NPC 8 controls	Analyzing set

GSE12452	*Cancer Epidemiology, Biomarkers & Prevention*	39	149	Illumina HiSeq 2000	21 NPC 10 controls	Validation set
GSE68799		644	55	Illumina Hiseq 2000	35 NPC 4 controls	Validation set

**Table 3:** Differentially expressed genes identified in verification sets.

Dataset	Upregulated genes	Downregulated genes
GSE68799	PTGS2, TNFAIP6	CR2
GSE12452	MMP1, PTGS2, TNFAIP6, MMP3	LTF, MSMB, UPK1B, SCGB1A1, CHST9, PROM1, CASC1, SPAG6, TEKT1, PIGR, CAPS, RRAD, MS4A1, FAM3D

## Data Availability

Original data we selected for analysis are available in the GEO database, and our reanalyzed data are available from the corresponding authors upon request.
